# Supplementary material for: Ankylosing spondylitis and kinesiophobia
Source: PeerJ. 2025 Feb 13;13:e19034. doi: 10.7717/peerj.19034 (PMC11830361; doi:10.7717/peerj.19034)
Supplement: Supplemental Information 2 [file peerj-13-19034-s002.docx]

**DATA FORM**

**Protocol number:**

**Age:**

**Gender:**

**Education status:**

**Job:**

**Marital status:**

**Disease duration:**

**BASDAI score:**

**BASFI score:**

**Medicines used:**

**Accompanying diseases:**

**Tampa kinesiophobia score:**
